# Management of Isolated Gastric Varices by Devascularization and Proximal Gastrectomy in Cirrhotic Patients

**DOI:** 10.1155/1994/50485

**Published:** 1994

**Authors:** Jan-Sing Hsieh, Che-Jen Huang, Tsung-Jen Huang

**Affiliations:** 1 Department of Surgery Kaohsiung Medical College Kaohsiung Taiwan; 2 Department of Surgery Kaohsiung Medical College 100 Shih-Chuan 1st Road Kaohsiung Taiwan

## Abstract

Twenty patients with liver cirrhosis were treated by surgery for bleeding from isolated gastric varices. The presence of tortuous and engorged gastric veins connecting with a large splenorenal shunt was demonstrated by transhepatic portography in all patients. The surgical procedures consisted of splenectomy, proximal gastrectomy, paragastric devascularization, and ligation of the splenorenal shunt. Sixteen patients survived the surgery. Four deaths were caused by emergency operation for uncontrollable hemorrhage in extremely poor risk patients. Of the 16 survivors, 15 had been followed wth endoscopy and portography for a mean period of 42 months. The other one died of hepatocellular carcinoma three years after surgery. There was no bleeding episode during the period of follow-up in these patients. Recurrent esophageal varices of mild degree were documented by endoscopy and portography in three patients. Portography demonstrated that several newly formed retroperitoneal veins arising from the junction of the portal and superior mesenteric veins joined to form recurrent varices in these three patients. There was no significant change of the mean portal venous pressure before and after surgery. Our data reveals that elective surgery may provide satisfactory results in patients with isolated gastric varices. Transhepatic portography is the method of choice in radiologic investigation for prominent gastric varices.


*HPB Surgery*, 1994, Vol. 7, pp. 201–209
Reprints available directly from the publisher
Photocopying permitted by license only

© 1994 Harwood Academic Publishers GmbH
Printed in the United States of America

### MANAGEMENT OF ISOLATED GASTRIC VARICES BY DEVASCULARIZATION AND PROXIMAL GASTRECTOMY IN CIRRHOTIC PATIENTS

JAN-SING HSIEH, CHE-JEN HUANG and TSUNG-JEN HUANG
*Department of Surgery, Kaohsiung Medical College, Kaohsiung, Taiwan*

*(Received 8 February 1993)*

Twenty patients with liver cirrhosis were treated by surgery for bleeding from isolated gastric varices. The presence of tortuous and engorged gastric veins connecting with a large splenorenal shunt was demonstrated by transhepatic portography in all patients. The surgical procedures consisted of splenectomy, proximal gastrectomy, paragastric devascularization, and ligation of the splenorenal shunt. Sixteen patients survived the surgery. Four deaths were caused by emergency operation for uncontrollable hemorrhage in extremely poor risk patients. Of the 16 survivors, 15 had been followed with endoscopy and portography for a mean period of 42 months. The other one died of hepatocellular carcinoma three years after surgery. There was no bleeding episode during the period of follow-up in these patients. Recurrent esophageal varices of mild degree were documented by endoscopy and portography in three patients. Portography demonstrated that several newly formed retroperitoneal veins arising from the junction of the portal and superior mesenteric veins joined to form recurrent varices in these three patients. There was no significant change of the mean portal venous pressure before and after surgery. Our data reveals that elective surgery may provide satisfactory results in patients with isolated gastric varices. Transhepatic portography is the method of choice in radiologic investigation for prominent gastric varices.

KEY WORDS: Liver cirrhosis, isolated gastric varices, transhepatic portography, devascularization and proximal gastrectomy

### INTRODUCTION

In patients with liver cirrhosis the development of esophagogastric varices has been well recognized. However, prominent gastric varices without esophageal varices which are termed isolated gastric varices (IGV) or fundal varices could be found in a small proportion of patients[1–3]. In such patients with IGV, portographic study usually delineates tortuous and engorged gastric veins with a large spontaneous splenorenal or gastrorenal shunt draining into inferior vena cava[4]. IGV represent a specific variety of collateral veins in cirrhotic patients. Bleeding from gastric varices is difficult to control by balloon tamponade, especially varices locating in the fundic area[5]. The experience and success of endoscopic sclerotherapy in achieving hemostasis of IGV is also limited[1,3,6]. Transhepatic occlusion of IGV is contraindicated because of the potential hazard of inadvertent embolization of adjacent organs via the splenorenal shunt. Surgery becomes necessary in most situations to control hemorrhage from IGV. Herein, we present our experience in dealing with the results of surgical management of IGV with special reference to the study of portography before and after surgery.

---

Address correspondence to: Jan-Sing Hsieh, MD, Department of Surgery, Kaohsiung Medical College, 100 Shih-Chuan 1st Road, Kaohsiung, Taiwan

### PATIENTS AND METHODS

### *Patients*

During the period between March 1983 and April 1990, 197 patients with portal hypertension were evaluated by transhepatic portography. Of them, isolated gastric varices were observed in 20(10%), combined esophageal and gastric varices in 76(39%), and esophageal varices alone in 101(51%) based on portographic findings. Of the 20 patients, there were 15 males and 5 females with ages ranging from 37 to 65 years. All the 20 patients had at least one episode of bleeding from gastric varices. All of them had portal hypertension and posthepatitic liver cirrhosis documented by clinical and histological examinations. Preoperative liver function was categorized according to Child's method and, grade A was found in 5, grade B in 11, and grade C in 4 patients. On admission, 6 of the 20 patients with IGV presented clinical features of hepatic encephalopathy and elevated serum ammonia. The degree of encephalopathy became more aggravated when the patient continued to bleed.

Every patient with bleeding gastric varices was given vasopressin soon after admission to our unit. Balloon tamponade was then applied if the patient continued to bleed for one hour after the introduction of vasopressin infusion. Endoscopic sclerotherapy and percutaneous transhepatic embolization were not tried because of the fear of systemic embolization through the large splenorenal shunt. Fifteen patients were able to control bleeding with these treatments. They got recovery from acute bleeding and had an elective surgery later. Unfortunately, five required an emergency procedure due to failure of our medical measures and uncontrollable hemorrhage.

### *Diagnostic Procedures*

Other specific studies included gastroduodenoscopy, transhepatic portography and pressure measurement. All patients had at least one episode of bleeding from gastric varices. The diagnosis of gastric varices without concomitant esophageal varices was first made with a gastroduodenoscopy (GIF P 10, Olympus Co., Tokyo, Japan). Subsequently the diagnosis was confirmed by detailed angiographic study. The technique for transhepatic portography has been described in more detail in the literature[4]. In all patients a selective splenic venogram was initially obtained to evaluate the patency of the splenic vein, the presence of gastric varices and the flow direction of the splenorenal shunt. Occasionally selective catheterization of the other portal branch and collateral veins was also performed. The portal pressure was measured when the tip of the catheter was in the main portal trunk. The puncture site in the midaxillary line was the reference point and the pressure was measured with a water manometer.

### *Surgical Therapy*

The surgical procedures for IGV included splenectomy, paragastric devascularization, proximal gastrectomy and pyloroplasty. After completion of splenectomy, proximal halves of the lesser and greater curvatures were completely devascularized. The splenorenal shunt was carefully identified, and then ligated and divided.

The stomach was resected 5 cm distal to the esophagogastric junction and the wound was closed with a stapler. A vertical gastrotomy was made to introduce the EEA surgical stapling instrument (Autosuture, USA) for reconstruction of the esophagus and stomach (Figure 1). The same surgical procedures were employed in all patients who were diagnosed to have IGV in this study.

### *Follow-up Studies*

The patients who have survived for more than one year after surgery had regular clinical follow-up including assessment of recurrent hemorrhage, encephalopathy and ascites etc. In addition, to investigate the effect of surgery on variceal recurrence and the change of portal venous pressure, postoperative endoscopy and portography were performed at least one year following surgery. At the follow-up portography, catheterization of the splenic vein was not feasible because it was ligated at the operation. Only superior mesenteric and portal venograms were taken. If there were newly formed collaterals demonstrated, they were selectively cannulated to assess the communication with esophagogastric varices.

### RESULTS

### *Clinical Outcomes*

Of the 15 patients who underwent elective surgery, there was no hospital mortality or morbidity related to anastomotic leakage. However, postoperative complications were encountered in four patients. Two patients had left subphrenic abscess

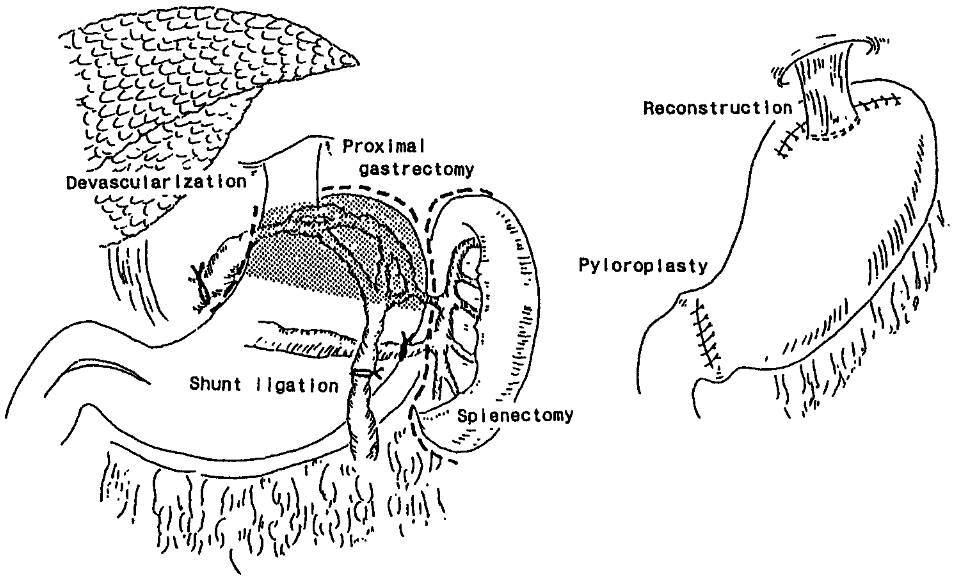

**Figure 1** Schematic drawing of the operative technique treating patients with isolated gastric varices.

due to extravasation of the pancreatic juice which was caused by pancreatic tail injury during splenectomy. They were cured within one month by intraperitoneal drainage. Two patients complained of dysphagia caused by mechanical stapling. The episode improved spontaneously without dilatation 6 months after surgery. Of the five patients who underwent emergency operation, two died 3 and 5 days after surgery, respectively. The causes of death were prolonged shock in one and respiratory failure in the other. Two developed progressive jaundice and died of hepatic failure within one month after surgery. All four patients were of Child's class C. In 4 of the 6 patients with hepatic encephalopathy, the episode did not attack again postoperatively. The other two who underwent emergency surgery died of progressive hepatic insufficiency finally. Of the 16 patients survived surgery, one died of hepatocellular carcinoma 3 years after surgery. The remaining 15 patients had been followed with endoscopy and portography. The mean follow-up period was 42 months ranging from 14 to 72 months, during which no patient had recurrent bleeding (Table I).

*Portographic Findings*

Transhepatic portography was successfully performed in all patients without any serious complications. When the tip of the catheter was positioned in the splenic vein near the hilum, injection of contrast medium demonstrated that the coronary and/or short gastric veins, arising from the splenic vein and forming varices in the proximal stomach, drained into the left renal vein and then into the inferior vena cava (Figure 2). We believe that such a communicating complex represents a characteristic angiographic appearance. A single and large venous channel, shunting substantial amount of blood from gastric varices into the left renal vein could be demonstrated in every one of our patients. In postoperative portography, neither the coronary vein nor other collaterals leading to the esophagogastric region were visualized in 12 patients (Figure 3). Three patients developed newly formed collaterals which originated from the junction of the portal and superior mesenteric veins. These new collaterals supplied several retroperitoneal veins which joined together and then drained into the esophagus (Figure 4).

*Portal Pressure*

Pressure studies confirmed the presence of portal hytal venous pressure before surgery in 20 patients was 34.3 ± 5.5 cm $H_2O$. In the 15 patients who had been followed up for portography after surgery, the mean portal venous pressure was 33.3 ± 4.7 cm $H_2O$. There was no significant difference between them.

**Table 1** Period of portographic and endoscopic follow-up

| *Period* | *No. of patients* | *No. of recurrence* |
|---|---|---|
| >1 Y (years) | 2 | 0 |
| >2 Y | 3 | 0 |
| >3 Y | 4 | 1 |
| >4 Y | 4 | 1 |
| >5 Y | 2 | 1 |

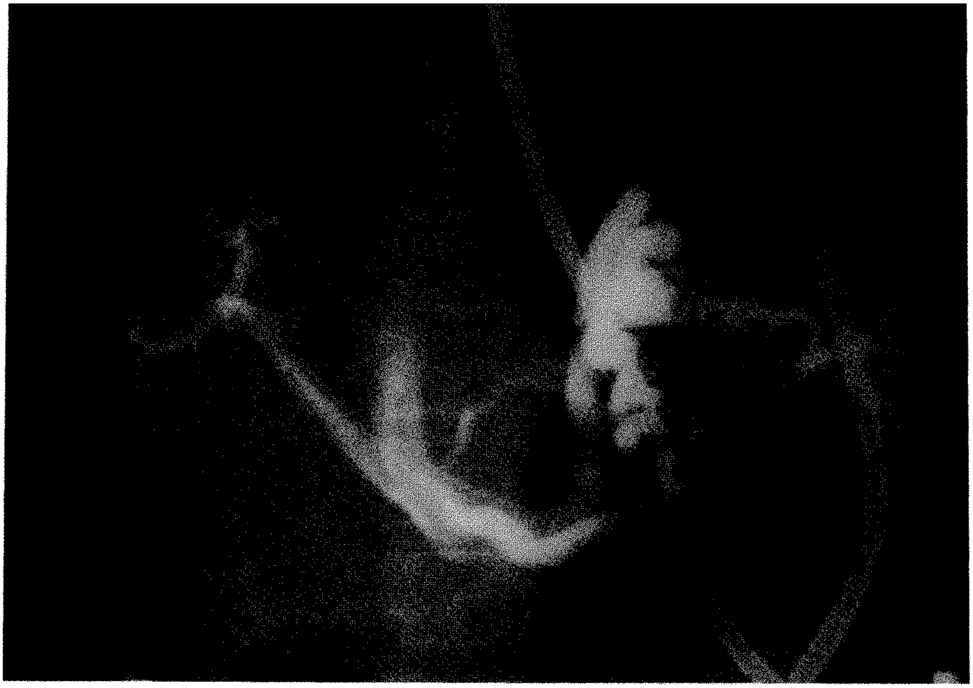

**Figure 2** Transhepatic portogram demonstrating isolated gastric varices with a splenorenal shunt draining into the left renal vein and inferior vena cava. No esophageal varices is found. Inflation of esophageal and gastric balloons is insufficient to prevent portal flow to gastric varices.

### *Endoscopic Findings*

In our patients, IGV usually appear as mass-like nodular and tortuous winding elevations of the mucosa in the cardia or fundus without the involvement of the esophagus at endoscopy. However, in three of these patients, IGV have been mistaken as submucosal tumor of the fundic stomach at preoperative endoscopy. Postoperatively, although three patients had endoscopically demonstrable esophageal varices, no recurrent gastric varices were verified. However, the degree of recurrent esophageal varices was mild in severity and they were obliterated by endoscopic sclerotherapy.

### DISCUSSION

In cirrhotic patients the mechanism for the development of gastric varices in the absence of esophageal varices remains poorly understood. The presence of IGV can be explained satisfactorily if splenic vein obstruction has occurred[7]. However, in patients with a patent splenic vein, it is difficult to account for the development of IGV. Vianna *et al.*[8], postulated that since the maximum resistance to flow from gastric varices to esophageal area is in the intrinsic veins at the palisade zone, gastric varices form proximal to this area. Felming and Seaman[9] stated that IGV

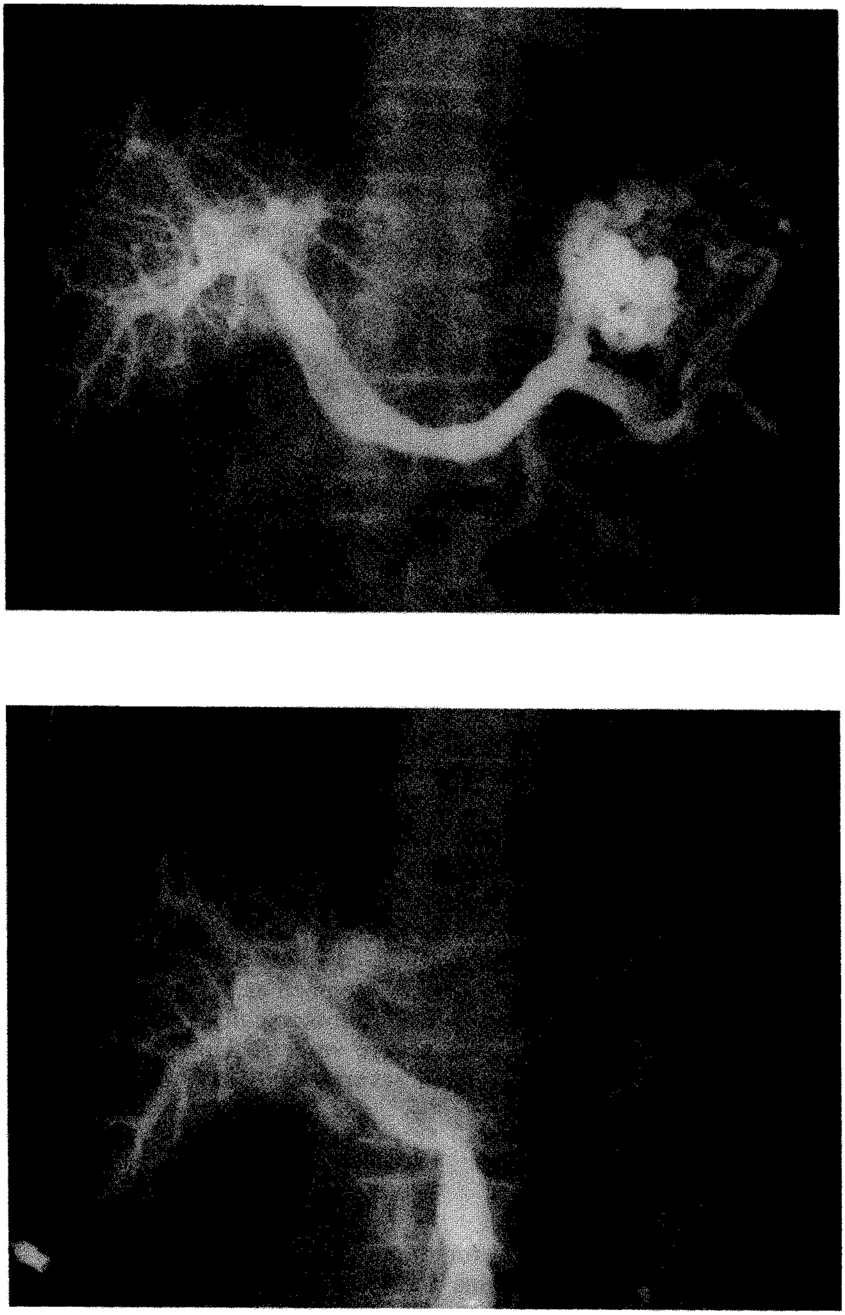

(A)

(B)

**Figure 3** Transhepatic portograms showing isolated gastric varices before surgery (A). Neither esophageal nor gastric varices is visible 5 years after surgery (B).

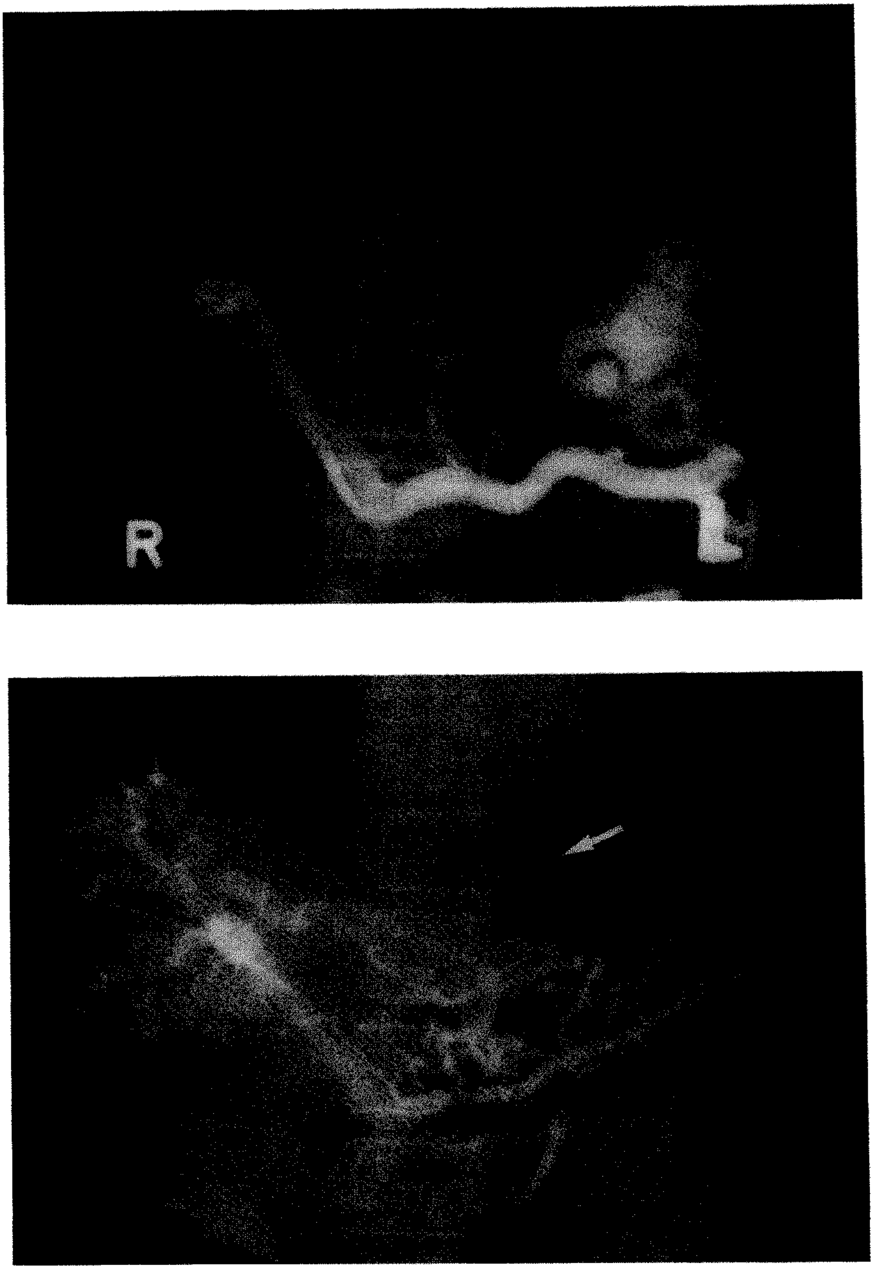

(A)

(B)

**Figure 4** Transhepatic portograms showing isolated gastric varices before surgery (A). A newly formed collateral arising from the bifurcation of the superior mesenteric and portal veins. Recurrent esophageal varices (arrow) of mild degree are demonstrated 4 years after surgery (B).

with a patent splenic vein may occur because of direct anastomoses between the gastric and retroperitoneal veins. Samuel[10] reported that dilatation of the fundal veins may antedate esophageal varices. In our opinion, preexisting venous channels confined to the fundic area may become dilated and lead to the development of gastric varices in response to portal hypertension. Halvorsen and Myking[11] found that the development of portosystemic shunts was not spontaneous, but rather represented dilatation of already existing vascular channels. When dilated venous channels (gastric varices) were eradicated by gastric resection, recurrent varices would not appear in the stomach but in the esophagus as demonstrated by postoperative portography in three of our patients. Our observation is in accord with that of the animal study[11].

Gastroduodenoscopy has been helpful in detecting gastric varices in most patients[1–4]. However, several reports revealed that IGV have been mistaken for neoplasms both radiologically and endoscopically[12,13]. We had such experience in three patients. Moreover, one should be careful in taking biopsy from a suspected submucosal lesion in cardiac and fundic regions in cirrhotic patients since it may cause massive and uncontrollable bleeding. In the present study, definite diagnosis was confirmed by transhepatic portography in all patients including three who had been misdiagnosed by endoscopy. Moreover, newly formed collaterals feeding recurrent varices could also be clearly demonstrated by this technique. We recommend that transhepatic portography and endoscopy should be used together for identification of IGV.

In this series the portal pressure is strikingly high in spite of the presence of a large splenorenal shunt. Furthermore, every patient had episodes of variceal bleeding. Our data are in accord with those of Lam and Aseni[14,15] that almost all cirrhotic patients with a large spontaneous portosystemic shunt showed high portal pressure and any protective effect on bleeding by the shunt is unpredictable.

The treatment of choice for the management of hemorrhage from IGV is controversial. Balloon tamponade with the Linton-Nachlas tube could achieve hemostasis with limited success[5]. The potential risk of emboli migration to the vital organs prevent the widespread use of percutaneous transhepatic embolization for IGV. Previous studies have shown that poor results of endoscopic sclerotherapy in treating gastric varices[1,3]. However, using improved sclerosing technique and agent, Soehendra *et al.*[16] have been able to successfully treat bleeding gastric varices and the results are encouraging. Previous reports[13,17–19] revealed that surgical treatments of IGV only limited to sporadic cases and most had inadequate follow-up. Surgical treatments had included ligation of the varices and two-thirds gastrectomy[17], splenectomy alone[13], esophagogastrectomy and splenectomy[13], proximal gastrectomy[18], and selective shunt surgery[19].

The rationale of our procedure in treating IGV is to interrupt directly all the varicose network. Resection of a segment of proximal stomach may reduce the immediate recannalization of venous collaterals and the extensive devascularization is to ablate as much as possible the extrinsic collaterals to the stomach. The purpose of splenectomy includes abolishing hypersplenism and eradicating the short gastric veins. Ligation of splenorenal shunt may prevent new development of retroperitoneal collaterals. Pyloroplasty may avoid gastric stasis due to the inevitable vagotomy during the procedure of extensive devascularization.

The late results in this small group presented here seem acceptable as compared to other types of direct surgical intervention[20]. The mortality was contributed mostly by emergency and poor-risk conditions. The surgical technique should not be recommended in emergency cases, since the management of acute bleeding from IGV is often difficult. Follow-up portography and endoscopy demonstrated that mild recurrent esophageal varices developed in three of 15 patients who survived surgery. These results also suggest that elective surgery may be the most definite approach for the management of IGV. Endoscopy and transhepatic portography provides the most direct methods for access of esophagogastric varices.

### *References*

1. Sarin, S.K., Sachdev, G., Nanda, R., Misra, S.P. and Broor, S.L. (1988) Endoscopic sclerotherapy in the treatment of gastric varices. *British Journal of Surgery*, **75**, 747–750
2. Greig, J.D., Garden, O.J., Anderson, J.R. and Carter, D.C. (1990) Management of gastric variceal haemorrhage. *British Journal of Surgery*, **77**, 297–299
3. Korula, J., Chin, K., Ko, Y. and Yamada, S. (1991) Demonstration of two distinct subsets of gastric varices. Observations during a seven-year study of endoscopic sclerotherapy. *Digestive Disease and Science*, **36**, 303–309
4. Watanabe, K., Kimura, K., Matsutani, S., Ohto, M. and Okuda, K. (1988) Portal hemodynamics in patients with gastric varices. A study in 230 patients with esophageal and/or gastric varices using portal catheterization. *Gastroenterology*, **95**, 434–440
5. Teres, J., Cecilia, A., Bordas, J.M., Rimola, A., Bru, C. and Rodes, J. (1978) Oesophageal tamponade for bleeding varices. Controlled trial between the Sengstaken Blakemore tube and the Linton Nachalas tube. *Gastroenterology*, **75**, 566–569
6. Trudeau, W. and Prindiville, T. (1985) Endoscopic injection sclerosis in bleeding gastric varices. *Gastrointestinal Endoscopy*, **32**, 264–268
7. Madsen, M.S., Petersen, T.H. and Sommer, H. (1986) Segmental portal hypertension. *Annals of Surgery*, **204**, 72–77
8. Viana, A., Hayes, P.C., Moscoso, G., Sriver, M., Portmann, B., Westaby, D. and Williams, R. (1987) Normal venous circulation of gastroesophageal junction. A route to understanding varices. *Gastroenterology*, **93**, 876–889
9. Fleming, R.J. and Seaman, W.B. (1968) Roentgenographic demonstration of unusual extra-esophageal varices. *American Journal of Roentgenology*, **103**, 281–290
10. Samuel, E. (1948) Gastric varices. *British Journal of Radiology*, **21**, 519–522
11. Halvorsen, J.F. and Myking, A.O. (1974) The porto-systemic collateral pattern in the rat. *European Surgical Research*, **6**, 183–195
12. Bachman, B.A. and Brady, P.G. (1984) Localized gastric varices: Mimicry leading to endoscopic misinterpretation. *Gastrointestial Endoscopy*, **30**, 244–247
13. Rice, R.P., Thompson, W.M., Kelvin, F.M., Kriner, A.F. and Garbutt, J.T. (1977) Gastric varices without esophageal varices. An important preendoscopic diagnosis. *Journal of the American Medical Association*, **237**, 1976–1979
14. Lam, K.C., Juttner, H.U. and Reynolds, T.B. (1981) Spontaneous portal-systemic shunt. Relationship to spontaneous encephalopathy and gastrointestinal hemorrhage. *Digestive Disease and Science*, **26**, 346–352
15. Aseni, P., Beati, C., Brambilla, G., Bertini, M. and Belli, L. (1986) Does large spontaneous portal systemic shunt in cirrhosis protect from the risk of gastroesophageal bleeding? *Journal of Clinical Gastroenterology*, **8**, 235–238
16. Soehendra, N., Grimm, H., Nam, V. Ch. (1990) Endoscopic obliteration of fundic varices. *Canadian Journal of Gastroenterology*, **4**, 643–646
17. Rossi, N.P., Cohen, C.N., Gulessarian, H. and Ehrenhaft, J.L. (1967) Isolated varices of the gastric fundus. *Annals of Surgery*, **165**, 640–643
18. Wohl, G.T. and Shore, F. (1959) Lesions simulating carcinoma of the cardiac end of stomach. *American Journal of Roentgenology*, **82**, 1048–1057
19. Warren, W.D., Millikan, W.J., Henderson, J.M., Rasheed, M.E. and Salam, A.A. (1984) Selective variceal decompression after splenectomy or splenic vein thrombosis. *Annals of Surgery*, **199**, 694–702
20. Inokuchi, K. (1985) Present status of surgical treatment of esophageal varices in Japan: A Nationwide survey of 3588 patients. *World Journal of Surgery*, **9**, 171–180

*(Accepted by S. Bengmark 25 April 1993)*

